# Suicidal ideation and attempt and associated factors among patients with substance use disorder: institution-based cross-sectional study

**DOI:** 10.1192/bjo.2022.551

**Published:** 2022-08-01

**Authors:** Gebeyaw Molla Kassie, Yohanes Mirekena Lemu, Mengesha Srahbzu Biresaw, Gebremeskel Mesafint Dessie, Getaneh Tesfaye Tadesse, Woredaw Minichil Gared, Mesele Wonde Belay

**Affiliations:** Department of Psychiatry, College of Health Science, Aksum University, Aksum, Ethiopia; Department of Psychiatry, School of Medicine, College of Medicine and Health Science, University of Gondar, Gondar, Ethiopia; Department of Nursing, College of Health Science, Mizan-Tapi University, Tapi, Ethiopia

**Keywords:** Suicidal behaviour, substance use disorder, Ethiopia

## Abstract

**Background:**

No published research in Ethiopia has examined the prevalence of suicidal ideation and suicide attempts and associated factors among patients with substance use disorder.

**Aims:**

The main aim of this study was to assess the prevalence of suicidal ideation, suicide attempt and associated factors among patients with substance use disorders.

**Method:**

An institution-based cross-sectional study was conducted from 5 May to 13 June 2019 in Addis Ababa. A total of 408 patients were identified using a systematic sampling technique. Data were collected through interviews using the suicidality module of the Composite International Diagnostic Interview. Data were entered into EpiData and analysed using SPSS. Logistic regression analyses were employed. Variables with *P* < 0.05 were considered to be statistically significant with 95% confidence intervals.

**Results:**

Prevalence rates of suicidal ideation and attempt were found to be 39.5% and 18.6%, respectively. Family history of mental illness (adjusted odds ratio (AOR) = 3.60, 95% CI: 2.17, 5.97), comorbid mental illness (AOR = 3.61, 95% CI: 2.11, 6.16), perceived stigma related to substance misuse (AOR = 4.00, 95% CI: 2.26, 7.07) and alcohol use (AOR = 7.49, 95% CI: 1.99, 28.19) were associated with suicidal ideation. Being female (AOR = 2.46, 95% CI: 1.08, 5.70), family history of suicide (AOR = 3.08, 95% CI: 1.68, 5.64), comorbid mental illness (AOR = 4.09, 95% CI: 2.23, 7.49) and khat use (AOR = 3.73, 95% CI: 1.24, 11.17) were associated with suicide attempt.

**Conclusions:**

The prevalence of suicidal ideation and attempt were both found to be high. In particular, patients who had a comorbid mental illness were at high risk of both suicidal ideation and attempt. Therefore, special attention should be given to those with a family history of suicide or comorbid mental illness.

Recent studies from various countries have indicated that patients with substance use disorder have a greater risk of suicidal ideation and suicide attempts compared with the general population.^1^ Alcohol use disorder was found to be associated with a tenfold increase in risk of dying by suicide in a meta-analysis of 42 different cohort studies; those who injected drugs were about 14 times more likely to complete suicide.^2^ Moreover, 58% of those with polysubstance dependence report suicide attempts.^3^ A study in Spain among patients who sought treatment for substance use disorder reported that the lifetime suicidal ideation rate was 43.7% and the lifetime suicide attempt rate was 17.7%, with 1 month prevalence of 8.7% and 1.5% for suicide attempt and ideation, respectively.^1^ The prevalence of suicidal ideation and attempt have also been reported by studies in different countries; for instance, 21% in Turkey for suicidal attempts,^4^ 34% and 13% in Australia for lifetime and recently attempted suicide among patients with heroin dependence,^5^ 44.7% for suicidal ideation and 26.8% for suicide attempts in the past 6 months in China,^6^ 6.3% for suicidal ideation and 2.1% for suicide attempt in Morocco^7^ and 20% for suicidal ideation over the past month in Kenya.^8^ The prevalence of suicidal ideation and attempts in Ethiopia has been reported among individuals with various diagnoses of mental disorders; 23.3% of those with a diagnosis of major depression, 23.8% of those diagnosed with bipolar disorder and 13.1% of those diagnosed with schizophrenia had made a suicide attempt.^9^ In another study from Ethiopia among adult psychiatric out-patients, rates of 64.8% and 19.2% for suicidal ideation and attempt were reported, respectively.^10^ There were multiple risk factors for suicidal ideation and attempt in patients with substance use disorders, including beginning use of a substance at a younger age, being female, being less educated, unemployment, and having a diagnosis of depression, bipolar disorder, anxiety or polysubstance use.^5,11–13^ No published research in Ethiopia has yet examined the prevalence of suicidal ideation and suicidal attempts and the associated risk factors among substance use disorder patients. This study aimed to fill this gap.

## Method

### Study design and setting

We followed the methods of Molla et al (2019).^14^ A cross-sectional study was conducted on patients with substance use disorder who visited the out-patient department of any of three public hospitals (Saint Amanuel Mental Specialised Hospital, Saint Paul's Millennium Medical College and Zewditu Memorial Hospital) in Addis Ababa, Ethiopia, from 5 May to 13 June 2019. Saint Amanuel Mental Specialised Hospital is the only mental health hospital in the country. The hospital has 300 beds, of which 14 are for substance use disorder patients. Saint Paul's Millennium Medical College provides psychiatry services and has 15 beds for substance use disorder patients. Zewuditu Hospital provides psychiatry services and has substance use disorder out-patient and in-patient services with five beds.

### Sample size determination and patient selection procedure

The total number of participants needed to conduct this study was calculated using a single population proportion formula with the following considerations: a standard normal distribution (*z* = 1.96) with 95% confidence interval (α = 0.05) and P = 50% (0.5), since there has been no study of the prevalence of suicidal ideation and attempt specifically in this population. The absolute precision or tolerable margin of error (*d*) was taken to be 5%. Ten per cent for non-respondents was added, bringing the total sample size to 423. Study participants from each hospital were allocated proportionally.

A systematic sampling technique was applied to select 423 patients with substance use disorders. The sampling interval (*k*) was calculated by dividing the total study population during the 1 month data collection period by the total sample size: *k* = *N* / *n* = 890 / 423 = 2.1. Therefore, participants were selected from patients every two intervals. The first study subject was selected by a lottery method from the first two participants. All patients with substance use disorder attending the out-patient department in the addiction psychiatry unit during the study period were included in the study. Those who were severely ill and difficult to interview were excluded from the study if they were not suitable owing to illness or if it was considered not to be in their best interests to participate.

### Outcome variables and independent variables

The outcome variables were suicidal ideation and attempt, assessed using the suicidality module of the World Mental Health Survey Initiative version of the World Health Organization's Composite International Diagnostic Interview (CIDI). There is a version of this assessment tool in the Amharic language, which has been validated for use in Ethiopia in both clinical and community settings.^15,16^

The study questionnaire had five components. Sociodemographic characteristics were collected using structured questions, clinical factors were collected using semi-structured questions, and substance-related factors were collected using substance-related questions. Social support was assessed using the Oslo three-item social support scale (Oslo-3). The total score on the Oslo-3 ranges from 3 to 14, indicating poor support (score of 3–8), moderate support (score of 9–11) or strong support (score of 12–14).^17^ Perceived stigma was assessed using the four-point Substance Abuse Perceived Stigma Scale (SAPSS). This is a 12-item Likert scale (strongly disagree, disagree, agree, strongly agree) that assesses the construct of perceived stigma.^18^

### Operational definition

#### Substance use disorder

Substance use disorder is the diagnostic term applied to specific substance misuse (e.g. alcohol use disorder or opioid use disorder) that results from the prolonged use of a substance, with a maladaptive pattern of substance use leading to clinically significant impairment or distress occurring within 12 months.^19^

#### Suicidal ideation

The respondents who answered ‘yes’ to the question ‘have you seriously thought about committing suicide within the past 1 year’ were considered to have suicidal ideation according to the suicidality module of CIDI.^20^

#### Suicidal attempt

The respondents who answered ‘yes’ to the question ‘have you attempted suicide within the past 1 year’ were considered to have had a suicide attempt according to the suicidality module of CIDI.^20^

#### Social support

The Oslo-3 social support scale was used to assess whether the participant had poor, moderate or strong support.^17^

#### Perceived stigma

Participants who scored less than or equal to the mean (≤24) on the 12-item SAPSS were considered to have perceived stigma related to substance misuse.^18^

#### Chronic medical illness

A participant was considered to have chronic physical illness if such an illness had previously been diagnosed and was currently being followed up.

#### Past and current mental illness

We noted previously and currently diagnosed mental illness and past and current treatment.

### Consent statement

Written informed consent was obtained from all participants.

### Data collection procedure

Data were collected by face-to-face interviews using the Amharic version of a pre-tested questionnaire and by reviewing the patient chart. The data was collected by three BSc psychiatry nurses who were supervised by two MSc mental health professionals. The supervisors and the data collectors were assigned to health institutions daily.

Two-day training for data collectors and supervisors was completed to ensure a common understanding of the interview questions. Ethical concerns that might be encountered during data collection, were discussed with the data collectors and supervisors. Pre-testing was conducted on 5%^21^ of the sample population at Eka-Kotebe General Hospital before the actual data collection to identify potential problems in using the data collection tools and to check the consistency of the data collectors. Data collected for the pre-test were not included in the analysis of the main study. Regular supervision was given by the supervisors and principal investigator to ensure that all necessary data were properly collected.

### Ethical statement

The authors assert that all procedures contributing to this work complied with the ethical standards of the relevant national and institutional committees on human experimentation and with the Helsinki Declaration of 1975, as revised in 2008. All procedures involving human subjects or patients were approved by the joint ethical review committee of the University of Gondar and Saint Amanuel Mental Specialised Hospital (reference UOG/AH/2365).

### Data processing and analysis

The collected data were checked for completeness and consistency and coded. The coded data were entered into a computer using EPI data version 3.1 and imported to SPSS for Windows version 20. Descriptive statistics (frequency, percentage and mean) were computed and presented using tables. Bivariate logistic analysis was performed to identify the variables associated with suicidal ideation. Variables with *P* < 0.2 in the logistic regression analysis were entered into the multivariable analysis to identify independent correlates of suicidal ideation. The Hosmer–Lemeshow test was used to determine the goodness of fit of the model. *P*-values less than 0.05 were considered to indicate statistical significance, and point estimates are presented with their respective 95% confidence intervals.

## Results

### Sociodemographic characteristics of the respondents

Of the total 423 patients, 408 participants were assessed and included in the analysis. The median age of participants was 31 years (interquartile range (IQR): 25–38), with age ranging from 18 to 70 years. The majority of the participants were males (352, 86.3%), 286 (70.1%) were single, 170 (41.7%) had completed high school education and 164 (40.2%) were unemployed (Table 1).

**Table 1:** Distribution of participants by sociodemographic factors (*N* = 408)

Sociodemographic variable	*n* (%)
Sex	
Male	352 (86.3%)
Female	56 (13.7%)
Religion	
Orthodox	268 (65.7%)
Muslim	48 (11.8%)
Protestant	80 (19.6%)
Other^a^	12 (2.9%)
Marital status	
Married	88 (21.6%)
Single	286 (70.1%)
Divorced or widowed	34 (8.3%)
Ethnic background	
Amhara	156 (38.2%)
Oromo	116 (28.4%)
Tigre	54 (13.2%)
Gurage	60 (14.7%)
Other^b^	22 (5.4%)
Education status	
No formal education or primary education	84 (20.6%)
Secondary and preparatory education	170 (41.7%)
Diploma	98 (24.0%)
Bachelor's degree or above	56 (13.7%)
Occupational status	
Government employee	50 (12.3%)
Student	76 (18.6%)
Merchant	20 (4.9%)
Private company employee	84 (20.6%)
Unemployed	164 (40.2%)
Other^c^	14 (3.4%)

a. Adventist or no religion.

b. Hadiya, Hadere, Welelle or Argoba.

c. Housewife, farmer or daily labourer.

### Clinical and substance-use-related factors of the respondents

The majority of participants used alcohol 377 (92.4%), and 16.4%, 17.9%, 29.9% and 35.8% of respondents used one, two, three and four types of substance, respectively; 136 (33.3%) of participants had a comorbid mental illness. The median (IQR) age of onset of substance use was 19 (16–23) years, and the duration of substance use was 10 (6–14) years. Of the total participants, 120 (29.4%) had a family history of suicide (Table 2).

**Table 2:** Description of clinical and substance-related factors (*N* = 408)

Variables	Frequency (%)
Family history of suicide	120 (29.4%)
Family history of mental illness	177 (43.4%)
Comorbid mental illness	136 (33.3%)
Comorbid physical illness	24 (5.9%)
Type of substance used	
Alcohol	377 (92.4%)
Khat	299 (73.3%)
Tobacco	318 (77.9%)
Cannabis	169 (41.4%)
Number of substances used	
One	67 (16.4%)
Two	73 (17.9%)
Three	122 (29.9%)
Four	146 (35.8%)
Median age at onset of substance use, years	19 (16, 23)
Median duration of substance use, years	10 (6, 14)
Median duration of substance use disorder treatment, months	3 (1, 8)

### Psychosocial factors

One-fifth of participants (20.10%) reported that they were getting strong support, and half (206, 50.5%) reported poor social support. The remaining 29.40% received moderate social support. More than half of the respondents (230, 56.37%) reported substance-related perceived self-stigma.

### Prevalence of suicidal ideation and attempt among patients with substance use disorder

Nearly half (198, 48.5%) of the respondents reported that they had experienced suicidal ideation at least once in their lifetime; 170 (85.85%) of these were men. Suicidal ideation in the past 12 months was reported by 39.5% of the participants; 83.85% of these were men.

A total of 118 (28.9%) respondents disclosed that they had attempted suicide at least once in their lifetime; 92.4% said they had planned an attempt. Nearly one in five patients (76, 18.6%) reported that they had attempted suicide in the past 12 months. Of the participants who had ever attempted suicide, 32(27.1%), 61(51.7%) and 25 (21.2%) of had attempted suicide once, twice and three or more times, respectively. The most commonly used method was reported to be hanging (65, 55.1%), followed by poisoning (32, 27.1%), and some participants had used more than one methods. The two most frequently mentioned major reasons for suicide attempt were family conflict (35, 29.7%) and poverty (27, 22.9%) (Table 3).

**Table 3:** Distribution of suicidal ideation and attempt (*N* = 408)

Variable	Frequency (%)
Lifetime suicidal ideation	198 (48.5%)
Suicidal ideation in past 12 months	161 (39.5%)
Lifetime plan of suicide	109 (26.7%)
Lifetime suicide attempt	118 (28.9%)
Suicide attempt in past12 months	76 (18.6%)
Frequency of suicide attempt	
Once	32 (27.1%)
Twice	61 (51.7%)
More than two times	25 (21.2%)
Reason for suicidal attempt	
Family conflict	35 (29.7%)
Poverty	27 (22.9%)
Financial loss	24 (20.3%)
Death in the family	11 (9.3%)
Mental illness	11 (9.3%)
Other^a^	10 (8.5%)
Method of attempt	
Hanging	65 (55.1%)
Poisoning	32 (27.1%)
Drug overdose	24 (20.3%)
Sharp tools	20 (16.9%)
Jumping from high places	8 (6.8%)

a. Hopelessness or no reason.

### Factors associated with suicidal ideation among patients with substance use disorder

The categorical variables (family history of suicide, family history of mental illness, comorbid mental illness, poor social support, perceived stigma related to substance misuse, alcohol use, khat use, tobacco use, cannabis use and multiple substance use) and continuous variables (age, age at onset of substance use, duration of substance use and duration of treatment) had *P*-values less than 0.2 for association with suicidal ideation in the bivariate analysis and were included in the subsequent multivariate analysis. In the multivariate analysis, family history of mental illness, comorbid mental illness, perceived stigma related to substance misuse and alcohol use had statistically significant associations with suicidal ideation.

Participants who had family history of mental illness had a nearly four times higher risk of having suicidal ideation compared with people with no family history of mental illness (adjusted odds ratio (AOR) = 3.60, 95% CI: 2.17, 5.97). Participants with comorbid mental illness were 3.61 times more likely to have suicidal ideation compared with patients who had no comorbid mental illness (AOR = 3.61, 95% CI: 2.11, 6.16). Patients reporting perceived stigma related to substance misuse were four times more likely to have suicidal ideation compared with those reporting no such stigma (AOR = 4.00, 95% CI: 2.26, 7.07). Alcohol use had a strong association with suicidal ideation. Respondents who used alcohol had a 7.49 times higher risk for suicidal ideation compared with those who did not use alcohol (AOR = 7.49, 95% CI: 1.99, 28.19) (Table 4).

**Table 4:** Associations between patient factors and suicidal ideation (*N* = 408)

Explanatory variable	COR (95% CI)	AOR (95% CI)	*P*-value
Family history of suicide			
Yes	2.48 (1.60, 3.83)	1.44 (0.84, 2.48)	
No	1	1	
Family history of mental illness			
Yes	3.78 (2.49, 5.75)	3.60 (2.17, 5.97)	0.000***
No	1	1	
Comorbid mental illness			
Yes	2.92 (1.91, 4.47)	3.61 (2.11, 6.16)	0.000***
No	1	1	
Social support			
Poor	1.72 (1.01, 2.92)	1.07 (0.54, 2.14)	
Moderate	0.83 (0.45, 1.51)	1.29 (0.62, 2.70)	
Strong	1	1	
Substance misuse perceived stigma			
Yes	3.31 (2.15, 5.08)	4.00 (2.26, 7.07)	0.000***
No	1	1	
Alcohol use			
Yes	3.67 (1.38, 9.77)	7.49 (1.99, 28.19)	0.003
No	1	1	
Khat use			
Yes	2.62 (1.60, 4.32)	1.63 (0.74, 3.56)	
No	1	1	
Tobacco use			
Yes	2.08 (1.24, 3.49)	0.92 (0.34, 2.45)	
No	1	1	
Cannabis use			
Yes	2.27 (1.51, 3.40)	1.59 (0.49, 5.14)	
No	1	1	
Number of used substances			
One	1	1	
Two	1.22 (0.56, 2.65)	0.73 (0.43, 1.57)	
Three	2.17 (1.10, 4.29)	1.13 (0.40, 3.21)	
Four	4.20 (2.17, 8.13)	1.31 (0.41, 4.21)	
Age, years	0.96 (0.94, 0.98)	0.95 (0.89, 1.01)	
Age at onset of substance use, years	0.95 (0.92, 0.98)	1.02 (0.95, 1.09)	
Duration of substance use, years	0.97 (0.94, 0.99)	1.00 (0.94, 1.06)	
Duration of treatment, months	0.98 (0.97, 1.00)	0.97 (0.94, 1.00)	

AOR, adjusted odds ratio; COR, crude odds ratio.

χ² = 3.88, d.f. = 8; Hosmer–Lemeshow test statistic, 0.86; reference value, 1.

***P < 0.001.

### Factors associated with suicide attempt among patients with substance use disorder

The categorical variables (being female, being single, family history of suicide, comorbid mental illness, use of three of four substances, alcohol use, khat use, tobacco use and cannabis use) and continuous variables (age, age at onset of substance use, duration of substance use and duration of treatment) had *P*-values less than 0.2 for association with suicide attempt in the bivariate analysis. These variables fulfilled the minimum requirements for inclusion in the multivariate analysis. Being female, family history of suicide, comorbid mental illness, duration of treatment and khat use were statistically significantly associated with suicide attempt (*P*-value less than 0.05) in the multivariate analysis. The odds of a suicide attempt among females were 2.46 times higher compared with males (AOR = 2.46, 95% CI: 1.08, 5.70).

Patients who had a family history of suicide were 3.08 times more likely to have had a suicidal attempt compared with those who had no family history of suicide (AOR = 3.08, 95% CI: 1.68, 5.64). Participants with comorbid mental illness were 4.09 times more likely to have had a suicidal attempt compared with patients who had no comorbid mental illness (AOR = 4.09, 95% CI: 2.23, 7.49). Patients who used khat had 3.73 times higher odds of suicide attempt compared with patients who did not use khat (AOR = 3.73, 95% CI: 1.24, 11.17). For each unit (1 month) increase in duration of treatment, the odds of suicide attempt decreased by 8% (Table 5).

**Table 5:** Associations between patient factors and suicide attempt (*N* = 408)

Explanatory variable	COR (95% CI)	AOR (95% CI)	*P*-value
Sex			
Male	1	1	
Female	1.74 (0.91, 3.35)	2.46 (1.08, 5.70)	0.031
Marital status			
Married	1	1	
Single	2.48 (1.17, 5.21)	2.16 (0.82, 5.70)	
Divorced/widowed	1.17 (0.33, 4.08)	2.95 (0.63, 13.67)	
Family history of suicide			
Yes	3.50 (2.09, 5.86)	3.08 (1.68, 5.64)	0.000***
No	1	1	
Comorbid mental illness			
Yes	3.35 (2.01, 5.59)	4.09 (2.23, 7.49)	0.000***
No	1	1	
Alcohol use			
Yes	3.54 (0.83, 15.18)	2.63 (0.43, 16.19)	
No	1	1	
Khat use			
Yes	4.37 (1.94, 9.84)	3.73 (1.24, 11.17)	0.019
No	1	1	
Tobacco use			
Yes	2.09 (1.03, 4.26)	0.91 (0.24, 3.34)	
No	1	1	
Cannabis use			
Yes	3.21 (1.91, 5.39)	1.82 (0.37, 8.88)	
No	1	1	
Number of used substances			
One	1	1	
Two	2.21 (0.64, 7.56)	1.53 (0.21, 3.35)	
Three	2.37 (0.76, 7.42)	1.00 (0.24, 4.17)	
Four	7.4 (2.56, 21.76)	0.69 (0.14, 3.29)	
Age, years	0.95 (0.91, 0.98)	1.04 (0.95, 1.13)	
Age at onset of substance use, years	0.95 (0.91, 0.99)	0.96 (0.87, 1.06)	
Duration of substance use, years	0.95 (0.91, 0.99)	0.93 (0.84, 1.02)	
Duration of treatment, months	0.95 (0.91, 0.99)	0.92 (0.86, 0.98)	0.013

AOR, adjusted odds ratio; COR, crude odds ratio.

χ² = 3.11, d.f. = 8; Hosmer–Lemeshow test statistic, 0.92; reference value, 1.

***P < 0.001.

## Discussion

The prevalence of suicidal ideation in our study was 39.5%. Our results regarding suicidal ideation were consistent with those of studies carried out in Spain (43.7%)^1^ and the USA (35.4%).^21^ Regarding suicidal ideation, we found a lower prevalence compared with those reported by studies carried out in China (44.7%),^6^ Barcelona, Spain (50%)^22^ and Chicago, USA (67%).^23^ A possible reason for the discrepancy may be related to study design; the study in Spain used retrospective cohorts,^22^ and the one in the USA was a case–control study.^23^ Another possible reason for the discrepancy might be differences in the number of substances included; cocaine and opiates users were also included in the study conducted in Spain.^22^ In addition, the China study used a different sampling method (snowball), a different study population (female and sex workers) and a different sample size (200), all of which may be reasons for the discrepancy.^6^

Our study found a higher prevalence of suicidal ideation compared with those reported by studies in Australia (13.0%),^5^ Mexico (22%), Catalonia, Spain (32.7%),^24^ Canada (8.0%)^25^ and the USA (19.1%).^26^ This discrepancy may have been due to differences in the type of study (a household community-based survey was conducted in Mexico) and in the study population (the USA study was among high school students).^26^ In addition, the study from Australia involved a single-substance user, which could explain the discrepancy.^5^ Furthermore, the USA study included those who had used substances at least once in their lifetime, which might be a source of discrepancy.^26^ The prevalence of suicide attempts in our study (18.6%) was in line with those reported by studies in Turkey (21%)^4^ and Spain (17.7%).^1^

Regarding factors, patients who had a family history of mental illness were more likely to have suicidal ideation than patients who had no family history of mental illness. This finding is supported by a study in Turkey.^4^ This association might be because children with mentally ill parents or family members might not receive good support and may be more likely to have a history of child maltreatment. This in turn may lead to poor mental health and suicidal behavior.^4^

In this study, comorbid mental illness was found to have significant association with suicidal ideation; this is also supported by previous studies conducted in Turkey,^4^ Spain^1^ and the USA.^26^ This association might be because of some mental illnesses like depression are more likely to think about suicide.^27,28^

Patients with perceived stigma related to substance misuse were more likely to have suicidal ideation than their counterparts. This finding is supported by previous studies done in China^6^ and New York, USA.^29^ The association might be due to patients with perceived stigma having low self-esteem, depressive symptoms, loneliness and a sense of dissatisfaction with social relationships, which heighten suicidal ideation.^30^ Alcohol use was strongly associated with suicidal ideation. Previous studies conducted in Spain^1^ and Canada^25^ reported similar findings regarding the association between alcohol use and suicidal ideation. This association might be due to the fact that alcohol use increases psychological distress and aggressive behaviour, including self-aggression, changes an individual's expectations and helps to propel or trigger suicidal ideation into action; it also constricts attention and inhibits effective coping strategies to avoid suicidal behavior.^31^

Regarding factors associated with suicide attempt, females were more likely to experience suicide attempt than males. This finding is supported by previous studies conducted in Australia,^5^ Spain^1^ and Turkey.^4^ The association might be due to the fact that females are more likely to disclose their difficulties than males. This might encourage them to attempt suicide.^22^ It might also be because females have a twofold greater risk of depression, which increases suicidal behaviour, compared with males.^27,28^

Patients who had a family history of suicide were more likely to attempt suicide compared with their counterparts. This result is supported by a study conducted in Turkey.^4^ A possible reason for this association is that individuals whose parents died by suicide might experience challenges related to child–parent separation and attachment, as well as socioeconomic insecurity, which in turn favour suicidal behavior.^32,33^

In this study, comorbid mental illness was significantly associated with suicide attempt. This result was in line with those of previous studies conducted in the USA,^26^ Australia,^5^ Spain^1^ and Turkey.^4^ This association might exist because individuals with certain mental illnesses (for instance, depression) are more likely to attempt suicide.^27^ It could also be due to the unpleasant feelings of experiencing public stigma towards mental illness.^34^

We also found that khat chewing was associated with suicide attempt. This finding is supported a review study of the adverse effects of khat previously conducted in England, which showed suicide attempt has been reported by several authors in the context of the khat withdrawal state.^35^ Our study result regarding khat chewing and the suicidal attempt has also been supported by another study conducted in Jimma high school students which reported as khat chewing has an association with suicidal ideation which might progress to suicidal attempt.^36^ This might also be because of evidences indicated as there is an association between khat chewing and mental distress.^37^

Furthermore, patients with a longer duration of treatment for substance use disorder were less likely to experience suicidal attempt compared with their counterparts. This finding is supported by the results of a study conducted in Australia.^5^ This association may exist because when patients receive treatment for an adequate time, they are more likely to develop the skills to cope with adverse life events. It may also be because depressive symptoms, which may increase suicidal behaviour, can be identified and treated earlier while patients are receiving treatment.^38^

### Limitation of the study

Although this study reports important findings in the under-investigated area of suicidal ideation and attempt in Ethiopia, there were some limitations that need to be considered before generalising from the results. The first limitation is the possibility of underreporting of events, owing to the data collection method and the nature of the conditions. There may have been some recall bias as a face-to-face interview method was used. There could also have been social desirability bias, as disclosing suicide is perceived as a socially sensitive issue in the community. We recommend follow-up studies to clarify the cause–effect relationships among certain factors, including the relationships of comorbid mental illness and perceived stigma related to substance misuse with suicidal ideation and attempt. We also recommend that future studies use data collection techniques other than interview (such as self-administered or web-based questionnaires) to avoid underreporting of substances used owing to fear of legal issues.

## Data Availability

The data that support the findings of this study are available from the corresponding author (M.S.B.) upon reasonable request.
