# Suicidal ideation and attempt and associated factors among patients with substance use disorder: institution-based cross-sectional study – CORRIGENDUM

**DOI:** 10.1192/bjo.2022.567

**Published:** 2023-01-20

**Authors:** Gebeyaw Molla Kassie, Yohannes Mirkena Lemu, Mengesha Srahbzu Biresaw, Gebremeskel Mesafint Dessie, Getaneh Tesfaye Tadesse, Woredaw Minichil Gared, Mesele Wonde Belay

**Keywords:** Suicidal behaviour, substance use disorder, Ethiopia

An author's name was misspelled in the above paper: Yohanes Mirekena Lemu should be Yohanes Mirkena Lemu. This has now been corrected in the online PDF and HTML copies of the article.

The authors apologise for the error.
